# Impact of accurate diagnosis of interstitial lung diseases on postoperative outcomes in lung cancer

**DOI:** 10.1007/s11748-022-01868-6

**Published:** 2022-08-23

**Authors:** Yoko Azuma, Susumu Sakamoto, Sakae Homma, Atsushi Sano, Takashi Sakai, Satoshi Koezuka, Hajime Otsuka, Naobumi Tochigi, Kazuma Kishi, Akira Iyoda

**Affiliations:** 1 Division of Chest Surgery, Department of Surgery, Toho University School of Medicine, 6-11-1 Omori-nishi, Ota-ku, Tokyo, 143-8541 Japan; 2 Division of Respiratory Medicine, Toho University School of Medicine, 6-11-1 Omori-nishi, Ota-ku, Tokyo, 143-8541 Japan; 3 Department of Surgical Pathology, Toho University School of Medicine, 6-11-1 Omori-nishi, Ota-ku, Tokyo, 143-8541 Japan

**Keywords:** Lung cancer, Interstitial lung disease, Air space enlargement with fibrosis

## Abstract

**Objective:**

The prognostic impact of interstitial lung disease (ILD) subclassification based on both high-resolution computed tomography (HRCT) scan findings and histopathological findings is unknown.

**Methods:**

We retrospectively analyzed 104 patients who were diagnosed with clinical ILD according to HRCT scan findings and who underwent lung cancer surgery. Via an expert multidisciplinary discussion, we re-classified HRCT scan findings and validated the histopathological patterns of ILDs in lung specimens.

**Results:**

There were several mismatches between HRCT scan findings and histological patterns. Moreover, 87 (83.7%) and 6 (5.8%) patients were diagnosed with definitive ILD and pathological non-ILD, respectively. Finally, 82 patients with idiopathic interstitial pneumonias (IIPs) were divided into the idiopathic pulmonary fibrosis (IPF) (*n* = 61) group and the other group (*n* = 21). The 5-year overall survival rate of the IPF group was significantly lower than that of the other group (22.8% vs 67.9%; *p* = 0.011). Sub-classification of IIPs was found to be an independent prognostic factor for overall survival in patients with lung cancer.

**Conclusion:**

An accurate diagnosis of IIPs/IPF according to both HRCT scan findings and histological patterns is important for providing an appropriate treatment among patients with lung cancer who presented with clinical ILD.

## Introduction

Interstitial lung disease (ILD) is characterized by a damaged lung parenchyma associated with different inflammatory and fibrotic patterns [1]. ILD has several subtypes with different pathophysiologies, and its treatment strategies are also diverse [1, 2]. Patients with ILD have a high rate of lung cancer and a high risk of fatal morbidities such as acute exacerbation (AE) of ILD associated with lung cancer treatment [3–6].

In particular, idiopathic interstitial pneumonias (IIPs) are a group of ILDs defined as diffuse parenchymal diseases with an unknown etiology. Idiopathic pulmonary fibrosis (IPF) is the most common subtype and is correlated with poor prognoses [7]. IPF is defined as a specific type of chronic, progressive fibrosing interstitial pneumonia. Moreover, the condition is associated with the histopathologic and/or radiologic patterns of usual interstitial pneumonia (UIP), and it occurs primarily in older adults, who have the worst prognosis among all ILD subgroups [8]. Therefore, an accurate diagnosis of ILD or its sub-classification is required for an appropriate lung cancer treatment and accurate prognostic prediction.

The American Thoracic Society (ATS), European Respiratory Society (ERS), Japanese Respiratory Society (JRS), and Latin American Thoracic Society (ALAT) updated the detailed diagnostic high-resolution computed tomography (HRCT) scan findings and histopathological patterns of IPF in 2018 [1]. However, previous reports about the surgical treatment of lung cancer with ILD have not provided a detailed ILD classification. Similarly, a comparison between the HRCT scan findings and histopathological patterns of IPF based on the 2018 guidelines has not been performed.

This study re-evaluated and compared the HRCT scan findings and histopathological patterns based on the 2018 guidelines for IPF in patients with lung cancer who underwent surgical resection and who were clinically suspected with ILD. This study is first report to validate the characteristics and prognosis of patients after surgery based on the ILD and IIP classifications in patients with lung cancer.

## Methods

The Ethics Committee of Toho University Omori Medical Center approved this retrospective study (M20122) on August 28, 2020. A written informed consent or opt-out consent was obtained from all patients.

### Study design and population

Data were initially obtained from patients with lung cancer who had undergone pulmonary resection at Toho University Hospital between January 2004 and December 2020. We included 104 patients diagnosed with ILD based on clinical data and radiographic images. The diagnostic criteria have been selected based on the examination period [1, 9, 10].

### Diagnostic criteria for patients with clinical ILDs

**Fig. 1:**
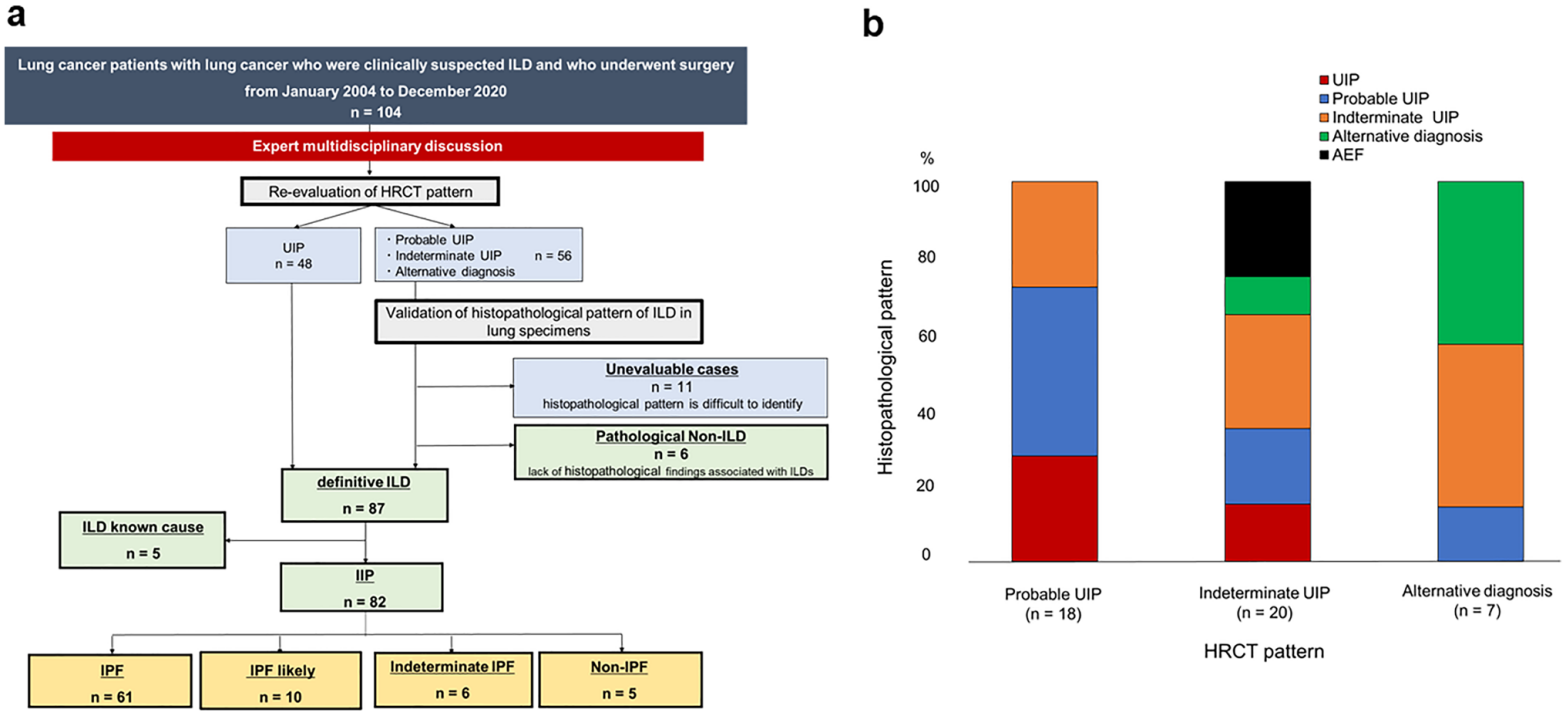
**a** Diagram of patient classification by diagnosis with HRCT and pathological findings **b** Comparison of HRCT scan findings and histopathological patterns in lung specimens in patients with patterns other than UIP on HRCT findings. *HRCT*: high-resolution computed tomography, *UIP*: usual interstitial pneumonia

Figure 1a shows the diagnosis and classification of patients. We re-classified the HRCT scan findings and validated the histopathological patterns of ILDs in lung specimens based on the latest 2018 diagnostic criteria by the ATS, ERS, JRS, and ALAT [1]. The diagnostic classification of ILDs was made via an expert multidisciplinary discussion between pulmonologists, thoracic pathologists, and radiologists. First, patients were divided into four subgroups according to HRCT pattern. Definitive ILD was defined as the presence of UIP pattern. Next, the histopathological pattern of patients with patterns other than UIP on HRCT scan was validated by assessing lung specimens. The patients were then divided into three groups: definitive ILD, pathological non-ILD, lack of histopathological findings associated with ILDs, and unevaluable, which is associated with histopathological patterns due to the excision range or lung specimen condition.

According to clinical data, patients diagnosed with definitive ILD were divided as follows: ILD with a known cause and IIPs. Finally, based on HRCT scan findings and histopathological patterns according to the 2018 IPF diagnostic criteria, patients with IIPs were divided into four groups, which are as follows [1]: IPF, IPF likely, indeterminate IPF, and non-IPF.

### Diagnostic criteria for airspace enlargement with fibrosis in lung specimens

The histopathological findings of airspace enlargement with fibrosis (AEF) were based on a previous report [11]. They are as follows: (1) fibrous interstitium with structural remodeling, (2) emphysematous change, (3) frequent bronchiolocentric location, and (4) absence of fibroblastic foci.

### Definition of AE

AE was defined according to the International Working Group Report [12] and the Japanese Respiratory Society [13] guidelines. Postoperative AE was defined as complications that developed within 1 month after surgery.

### Statistical analysis

The two groups were compared using the student’s *t*-test for variables with a normal distribution or the Mann–Whitney *U* test for variables with a non-normal distribution. Survival curves were prepared using the Kaplan–Meier method, and a univariate comparison was performed using the log-rank test. Multivariate analysis was performed using the Cox proportional hazards model. All statistical analyses were performed using JMP version 14.0 (SAS Institute Inc., Cary, NC, the USA).

## Results

This study enrolled 104 patients with clinical ILD, including those suspected with the condition and those who underwent surgical resection for lung cancer. Supplementary Table 1 shows the characteristics of all patients. The mean age of the patients was 71.2 years, and majority were men and had a history of smoking. In total, 79 (76.0%) patients underwent lobectomy. The most common histological type of lung cancer was squamous cell carcinoma (51.0%), and 54 (51.9%) patients were diagnosed with pathological stage 1 disease. On HRCT, 48 (46.2%) patients presented with a UIP pattern, 24 (23.1%) with a probable UIP pattern, 25 (24.0%) with an indeterminate UIP pattern, and 7 (6.7%) with an alternative diagnosis pattern.

### Mismatch between HRCT scan findings and histopathological patterns

We compared the HRCT scan findings and the histopathological patterns of ILDs in patients with patterns other UIP on HRCT scan using evaluable resected lung specimens (*n* = 45). The lung specimens of 11 patients were not suitable for the evaluation of ILDs because their specimens did not include sufficient fibrotic or noncancerous areas. Most patients (90.9%) underwent surgery of the upper lung lobe.

Figure 1b depicts the percentage of histopathological patterns for each HRCT scan finding. Patients with an alternative diagnosis on HRCT scan did not exhibit a histopathological UIP pattern. Patients with indeterminate UIP pattern on HRCT scan presented with all histopathological patterns at different proportions. Notably, some patients without histopathological characteristics of ILD presented with indeterminate UIP or an alternative diagnosis pattern on HRCT scan. The lung specimens showed pathological AEF among, and patients with this finding were included in the pathological non-ILD group (*n* = 6).

### Similarities and differences in HRCT scan and lung specimen findings between patients diagnosed with IIP and those with pathological non-ILD

**Fig. 2:**
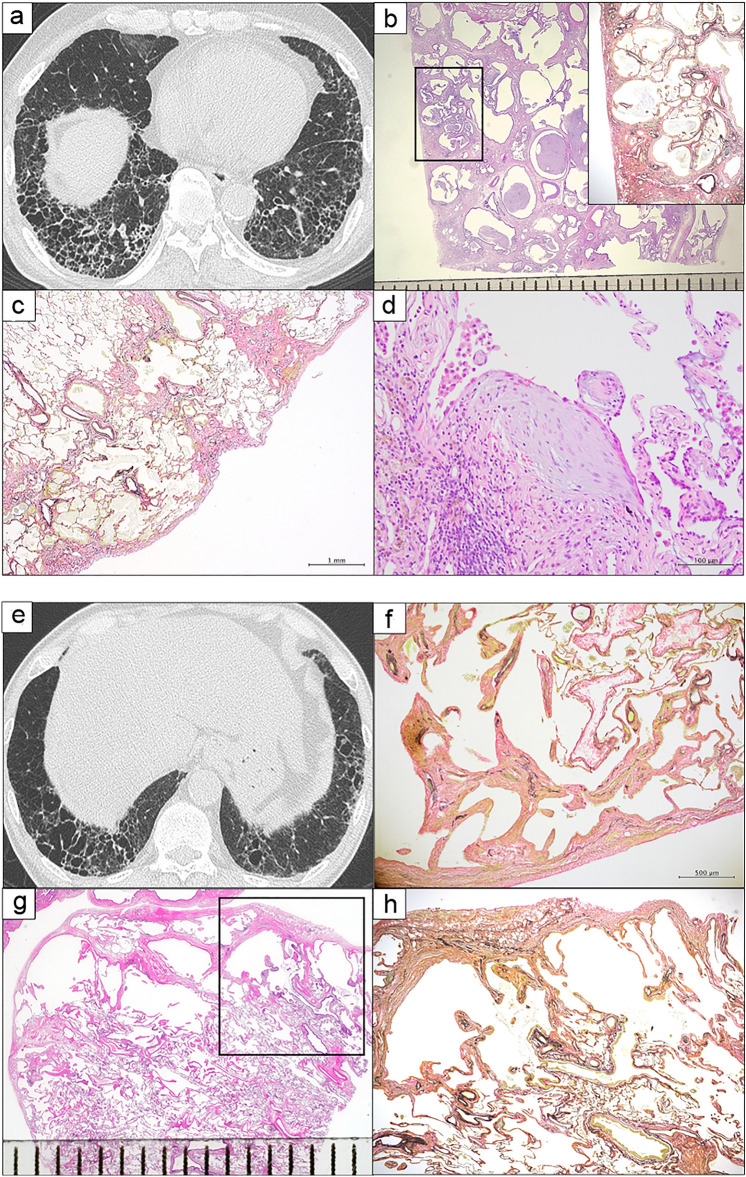
Comparison of HRCT scan findings and histopathological patterns between patients diagnosed with IIP and those with pathological non-ILD. **a**–**d**. An IIP case. **a** The UIP pattern on HRCT: presence of honeycombing with sub-pleural and basal predominance. **b** Panoramatic view (scale bar: 1 mm) and low-magnification photograph (box) (Elastica van Gieson staining) showing dense fibrosis with architectural distortion in the form of honeycomb change. **c** Low-magnification photomicrograph showing predominant sub-pleural and para-septal distribution of fibrosis (scale bar: 1 mm, **H** & **E**). **d** Higher-magnification photomicrograph showing fibroblast foci (scale bar: 100 µm, **H** & **E**).**e**–**h**. A pathological non-ILD case. **e** Indeterminate UIP pattern on HRCT: multiple thin-walled cysts in the lower lobe. **f** High magnification photomicrograph showing mild fibrosis with centriacinar emphysema in the background (scale bar: 500 µm, Elastica van Gieson stain). **g** Panoramatic view (scale bar: 1 mm) and **h** low-magnification photomicrograph (box in Fig. 4 g) (Elastica van Gieson staining) showing a fibrous wall of bronchiolocentric cysts. *HRCT*: high-resolution computed tomography, *IIP*: idiopathic interstitial pneumonia, *ILD*: interstitial lung disease, *UIP*: usual interstitial pneumonia

We compared the HRCT scan findings and histopathological patterns between patients diagnosed with IIP and those with pathological non-ILD, as shown in Fig. 2, respectively. Figure 2a shows the UIP pattern on HRCT scan image (presence of honeycombing with sub-pleural and basal predominance). Furthermore, histopathological findings including dense fibrosis with architectural distortion in the form of honeycomb change (Fig. 2b), predominant sub-pleural and para-septal distribution of fibrosis (Fig. 2c), and presence of fibroblast foci (Fig. 2d) were the characteristics of a typical UIP pattern. In contrast, a patient diagnosed with pathological non-ILD presented with multiple thin-walled cysts in the lower lung lobe, which is referred to as the indeterminate UIP pattern on HRCT scan (Fig. 2e). Moreover, this patient exhibited the characteristic histopathological findings of AEF: mild fibrous interstitium and emphysematous change without fibroblastic foci (Fig. 2f) and fibrous wall of bronchiolocentric cysts (Figs. 2g , h). Five (83.3%) patients in the pathological non-ILD group had AEF.

### Clinicopathological characteristics and prognoses between the IIP and other groups

Patients diagnosed with definitive ILD according to HRCT scan findings and histopathological patterns were classified into the IIP (*n* = 82) and ILD with a known cause (*n* = 5) groups. To assess the prognostic impact of IIP diagnosis, we compared the characteristics of patients and postoperative prognoses between the IIP and other groups. Table 1 shows the characteristics of the two groups.

**Table 1:** Characteristics between the IIP and other groups

	IIP (*n* = 82)	Pathological non-ILD (*n* = 6)	*p* value*	ILD with a known cause (*n* = 5)	*p* value**
**Male sex**	73 (89.0)	4 (66.7)	0.110	0	< 0.001
**Smoking history, yes**	79 (96.3)	6 (100)	0.634	2 (40.0)	< 0.001
**Tobacco, pack-years**	50.7 ± 32.8	69.4 ± 34.2	0.183	13.4 ± 21.0	0.014
**KL-6 level (U/mL)**	641.4 ± 371.7	521.3 ± 298.3	0.442	916.2 ± 443.1	0.116
**SP-D level (ng/mL)**	150.1 ± 93.5	106.9 ± 64.2	0.271	129.8 ± 38.7	0.632
**Pulmonary function**					
VC%pred (%)	98.6 ± 18.6	108.1 ± 22.4	0.235	101.8 ± 20.3	0.708
DLco%pred (%)	73.8 ± 20.7	72.0 ± 28.8	0.842	61.9 ± 22.5	0.216
FEV_1_/FVC (%)	75.5 ± 8.4	60.3 ± 8.1	< 0.001	78.8 ± 7.9	0.396
**HRCT pattern**			< 0.001		< 0.001
UIP	47 (57.3)	0		1 (20.0)	
Probable UIP	18 (22.0)	0		0	
Indeterminate UIP	13 (15.9)	6 (100)		1 (20.0)	
Alternative diagnosis	4 (4.9)	0		3 (60.0)	
**Surgical procedure**			0.569		0.707
Pneumonectomy	1 (1.2)	0		0	
Lobectomy	61 (74.4)	6 (100)		3 (60.0)	
Segmentectomy	4 (4.9)	0		0	
Partial resection	16 (19.5)	0		2 (40.0)	
**Clinical staging**			0.684		0.368
I	41 (50.0)	2 (33.3)		4 (80.0)	
II	24 (29.3)	2 (33.3)		1 (20.0)	
III	17 (20.7)	2 (33.3)		0	
**Pathologic staging**			0.425		0.516
I	41 (50.0)	2 (33.3)		4 (80.0)	
II	17 (20.7)	3 (50.0)		1 (20.0)	
III	22 (26.8)	1 (16.7)		0	
IV	2 (2.4)	0		0	
**AE from any cause**	24 (29.3)	0	0.120	1 (20.0)	0.657
Postoperative AE	13 (15.9)	0	0.291	0	0.334
**Adjuvant therapy**			0.906		0.838
Carboplatin-based	8 (9.8)	1 (16.7)		0	
Cisplatin-based	1 (1.2)	0		0	
Other	3 (3.7)	0		0	

Data were presented as n (%) or mean ± SD. *IIP*: idiopathic interstitial pneumonia, *ILD*: interstitial lung disease.

*Significance of IIP vs non-ILD.

**Significance of IIP vs ILD with a known cause

*KL-6*: Krebs von Den Lungen-6, *SP-D*: pulmonary surfactant protein-D, *VC%pred*: percentage of predicted vital capacity, *DLco%pred*: percentage of predicted diffusing capacity of the lungs for carbon monoxide, *FEV*_*1*_: forced expiratory volume in 1 s, *FVC*: forced vital capacity, *PaO*_*2*_: partial pressure of oxygen, *HRCT*: high-resolution computed tomography, *UIP*: usual interstitial pneumonia, *AE*: acute exacerbation

The pathological non-ILD group had a significantly lower forced expiratory volume in 1 s/forced vital capacity (FEV_1_/FVC) ratio at the initial visit than the IIP group (*p* < 0.001). On HRCT scan, all patients in the pathological non-ILD group had an indeterminate UIP pattern. There was a significant difference in HRCT patterns between the IIP and pathological non-ILD groups (*p* < 0.001). In the IIP group, 24 (29.3%) patients developed AE. Among them, 13 (15.9%) had AEs postoperatively. By contrast, patients with pathological non-ILD did not develop AEs during the study period.

The sub-classifications were microscopic polyangiitis-associated ILD in three and collagen vascular disease-associated ILD in two patients in group with ILD with a known cause. The proportion of women and never smokers was significantly higher in patients with ILD with a known cause than those with IIP (*p* < 0.001). In total, 13 (15.9%) patients in the IIP group developed postoperative AEs, but none in the ILD with a known cause group.

**Fig. 3:**
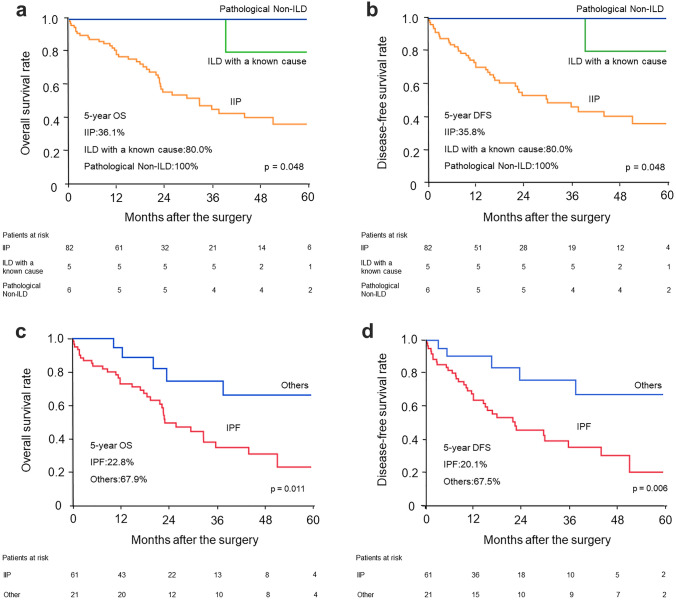
**a** Overall survival rate and **b** Disease-free survival rate of patients with IIP, ILD with a known cause, and pathological non-ILD. **c** Overall survival rate and **b** Disease-free survival rate of patients with IPF and other IIP groups. *IIP*: idiopathic interstitial pneumonia, *ILD*: interstitial lung disease, *IPF*: idiopathic pulmonary fibrosis

Patients in the pathological non-ILD and ILD with a known cause groups had a longer 5-year overall survival rate (OS) and disease-free survival rate (DFS) than the IIP group (*p* = 0.048, *p* = 0.048) (Fig. 3a , 3b).

### Clinicopathologic findings and prognoses between the IPF and other IIP groups

Next, patients with IIP were classified into three groups according to the 2018 IPF diagnostic guidelines. The groups were as follows: IPF (*n* = 61), probable IPF or indeterminate IPF (*n* = 16), and non-IPF (*n* = 5). The cumulative 5-year OS rates were 22.8% in the IPF group, 63.7% in the likely IPF or indeterminate IPF group, and 83.3% in the non-IPF group. The IPF group had a significantly worse OS than the likely IPF and indeterminate IPF groups (*p* = 0.023).

Finally, we divided patients with IIP into the IPF (*n* = 61) and other (*n* = 21) groups. Table 2 shows the characteristics of patients. The IPF group more commonly presented with AE from any cause than the other group (*p* = 0.021). Further, the 5-year OS and DFS of all patients with IPF were significantly worse than the other group (22.8% vs 67.9%, *p* = 0.011, 20.1% vs 67.5%, *p* = 0.006) (Fig. 3c , 3d). Supplementary Table 2 shows the causes of death in patients with IIP. AE was the most frequent cause of death in the IPF group. Nevertheless, none of patients died from AE in the other group.

**Table 2:** Characteristics of patients with IIPs

	Patients with IIP (*n* = 82)		
	Patients with IPF	Others	*p* value
	*n* = 61	*n* = 21	
**Age**	70.7 ± 6.6	71.1 ± 8.0	0.727
**Male sex**	54 (88.5)	19 (90.5)	0.805
**Tobacco, pack-years**	49.2 ± 24.8	54.8 ± 48.6	0.495
**VC%pred (%)**	96.5 ± 20.1	104.6 ± 11.7	0.084
**DLco%pred (%)**	71.7 ± 21.1	79.8 ± 18.7	0.121
≥ 80%	22 (36.1)	11 (52.4)	0.189
< 80%	39 (63.9)	10 (47.6)	
**KL-6 level (U/mL)**	684.1 ± 403.8	527.0 ± 239.3	0.091
**GAP staging, I/II**	47 (77.0)/14 (23.0)	20 (95.2)/1 (4.8)	0.063
**Comorbidities**			
COPD	20 (32.8)	10 (47.6)	0.224
Cardiovascular disease	33 (54.1)	12 (57.1)	0.809
Diabetes mellitus	17 (27.9)	5 (23.8)	0.717
**Surgical procedure**			0.886
Pneumonectomy	1 (1.6)	0	
Lobectomy	46 (75.4)	15 (71.4)	
Segmentectomy	3 (4.9)	1 (4.8)	
Partial resection	11 (18.0)	5 (23.8)	
**Histology**			0.235
Adenocarcinoma	17 (27.9)	11 (52.4)	
Squamous cell carcinoma	32 (52.5)	7 (33.3)	
Small cell carcinoma	5 (8.2)	1 (4.8)	
Others	7 (11.5)	2 (9.5)	
**Clinical staging**			0.391
I	30 (49.2)	11 (52.4)	
II	20 (32.8)	4 (19.0)	
III	11 (18.0)	6 (28.6)	
**Pathologic staging**			0.665
I	29 (47.5)	12 (57.1)	
II	14 (23.0)	3 (14.3)	
III	17 (27.9)	5 (23.8)	
IV	1 (1.6)	1 (4.8)	
**AE from any cause**	22 (36.1)	2 (9.5)	0.021
Postoperative AE	12 (19.7)	1 (4.8)	0.286
**Adjuvant chemotherapy**			0.778
Carboplatin-based	5 (8.2)	3 (14.3)	
Cisplatin-based	1 (1.6)	0	
Other	2 (3.3)	1 (4.8)	
**Recurrence, present**	20 (32.8)	8 (38.1)	0.658

Data were presented as *n* (%) or mean ± SD; *IIP*: idiopathic interstitial pneumonia, *IPF*: idiopathic pulmonary fibrosis, *VC%pred*: percentage of predicted vital capacity, *DLco%pred*: percentage of predicted diffusing capacity of the lungs for carbon monoxide, *KL-6*: Krebs von Den Lungen-6, *GAP*: gender–age–physiology, *COPD*: chronic obstructive pulmonary disease, *AE*: acute exacerbation

### Univariate and multivariate analyses of the prognostic factors for OS

Based on the univariate analysis of patients with IIPs, IIP classification, VC%pred, DLco%pred, and clinical stage were the prognostic factors for long-term prognosis. According to the multivariate analysis, IIP classification and clinical stage were considered independent prognostic factors for OS (Supplementary Table 3).

## Discussion

This study first revealed that patients with lung cancer diagnosed with clinical ILD and those with pathological non-ILD had different ILD classifications. Histopathological examination confirmed the mismatch between HRCT scan findings and pathological patterns and the prognostic impact of an accurate ILD and IIP sub-classification in patients with lung cancer.

In patients with lung cancer, the coexistence or classification of ILDs has a significant influence on lung cancer treatment and prognosis. Chemotherapy and radiation therapy are correlated with a high incidence of AE (10%–25% and 12.5%, respectively) [4–6]_._ Therefore, surgical resection is the only treatment option for lung cancer. However, postoperative patients with ILDs are at risk of AE. Based on a large multi-institutional cohort study of patients with ILD and non-small cell lung cancer who underwent surgery, the incidence of postoperative AE was 9.3%. Moreover, about 43.9% of patients died of this complication [6].

In terms of ILD classifications, patients with IPF have a high incidence of AE associated with lung cancer treatment, and IPF itself is a progressive disease with a poor prognosis. Previous reports showed that 10.7%–23.1% of patients with IPF developed postoperative AE [14, 15] and IPF was a risk factor for AE after chemotherapy [16]. Our data showed that an accurate classification of ILD and IIP affects prognosis after lung cancer surgery.

The diagnosis and classification of ILDs often require a multidisciplinary discussion between pulmonologists, radiologists, and pathologists with experience in the field of interstitial lung diseases [17]. In particular, in patients clinically suspected with IPF with HRCT scan findings, such as probable UIP, indeterminate UIP, and alternative diagnosis, surgical lung biopsy is recommended for the definitive diagnosis of IPF [1]. In fact, this study showed several mismatches between HRCT scan findings and histopathological patterns.

In particular, it is challenging to distinguish UIP from AEF based on HRCT scan findings alone. IPF and AEF are considered smoking-related lung abnormalities, and both often represent a multiple cyst resembling a honeycomb on HRCT scan images among heavy smokers [18–21]. By contrast, in most cases, the clinical course of patients with AEF alone is stable [18], and there is no risk of AE. In this study, almost all patients diagnosed with pathological non-ILD presented with AEF and concurrent COPD. Their prognosis was better than that of patients with IIPs, and none developed AE from any cause.

As mentioned above, the definitive diagnosis of lung abnormalities in patients with

lung cancer who were clinically suspected with ILDs has important implications in both lung cancer treatment and ILD management. Extraction of pathological non-ILD patients can allow them to receive the benefits of lung cancer treatment without limitations. By contrast, an early definitive diagnosis of ILDs can facilitate cautious management and provide appropriate interventions with anti-fibrotic drugs or prednisolone. In this study, the lung specimens obtained via upper lobe or partial resection were not eligible for obtaining a pathological ILD diagnosis. When performing surgical procedures, simultaneous surgical lung biopsy at the appropriate site should be considered for the diagnosis of ILD except among patients with a definitive UIP pattern on HRCT scan.

Since surgical treatment is often the only treatment for ILD-related lung cancer, the most effective surgical method should be selected regardless of ILD classification, and cardiopulmonary function and comorbidities should remain criteria for surgical selection. With regard to postoperative adjuvant chemotherapy, patients diagnosed with ILD by imaging or histopathology are at risk of acute exacerbation, regardless of ILD classification, and the indication for adjuvant chemotherapy should be carefully determined. However, in cases such as AEF that are not histologically ILD, postoperative adjuvant chemotherapy should be aggressively considered.

This retrospective study had several limitations and biases. The number of patients with ILD with a known cause or pathological non-ILD was limited.

## Conclusion

An accurate, detailed diagnosis of IIP/IPF according to both HRCT scan findings and histopathological patterns is important for providing an appropriate treatment among patients with lung cancer.
